# Clinical Profile of *Plasmodium vivax* Malaria in Children and Study of Severity Parameters in relation to Mortality: A Tertiary Care Centre Perspective in Mumbai, India

**DOI:** 10.1155/2014/765657

**Published:** 2014-11-02

**Authors:** Manju Kumari, Radha Ghildiyal

**Affiliations:** Department of Pediatrics, T. N. Medical College, BYL Nair Hospital, Mumbai, India

## Abstract

*Background*. While research on *P. vivax* is scarce because it is considered benign, it has become evident with implementation of molecular diagnosis that it can also cause multiple organ dysfunction and severe life-threatening disease. *Objective*. To study clinical presentations and complications of *P. vivax* malaria and mortality correlation to severity parameters as defined by WHO criteria for severe malaria. *Materials and methods*. This study was conducted in a tertiary care centre in Mumbai. Confirmed *P. vivax* cases were enrolled and studied for their clinical profile, and WHO severity parameters were tested for their frequency and association to mortality. *Result*. The most common presentation was fever followed by pallor. 26% of the cases satisfied one or more criteria of WHO severity parameters. 2 cases died; both had pulmonary edema and bleeding. The major predictor of mortality among these predefined severity criteria was pulmonary edema/ARDS. Patients with severe anemia, circulatory collapse, and repeated generalized convulsion had 100% survival rate. Leukopenia was present in 10% of the cases. Both cases with mortality had leukopenia. *Conclusion*. *P. vivax* monoinfection tends to have severe complications in children. There is a need to review severity criteria for *P. vivax* malaria.

## 1. Introduction

Four countries account for more than 80% of estimated cases of* P. vivax* cases (Ethiopia, India, Indonesia, and Pakistan). India alone contributes 80% of southeast Asia malaria burden. The risk of severe* P. vivax* disease in residents of endemic areas has been observed to rise with increasing transmission intensity, although the contribution of less access to care and more comorbidity in these settings is not well quantified. As a result of the slower rate of decrease in the incidence of* P. vivax*, many malaria control programs that are moving towards elimination need to give greater attention to the control of* P. vivax*, particularly in countries outside sub-Saharan Africa. Indeed,* P. vivax* predominates in countries in the preelimination and elimination phases [1].

*P. vivax *malaria has long been considered to have a benign course with multiple relapses. The typical complications seen in* P. falciparum *malaria are not usually found in* P. vivax* monoinfections. However, during the past few years, the trend in the clinical manifestations of* P. vivax *malaria has been changing [2]. Several isolated studies from India have reported severe complicated cases of* P. vivax *malaria [3].

Although this type of malaria is an enormous burden of disease, research about the disease is scarce probably because of it being considered benign malaria compared to* P. falciparum* malaria. However, with implementation of molecular diagnosis, it has become evident that* P. vivax* monoinfection could also be involved in multiple organ dysfunction and severe life-threatening disease as seen in* P. falciparum* infection [4, 5].

## 2. Materials and Methods

### 2.1. Setting of Study

A prospective hospital-based clinical observational study was done over 2 years in a pediatric ward of a tertiary care hospital situated in Mumbai, India. A total of 50 patients under age of 12 years were enrolled in the study.

### 2.2. Ethical Issues

The institutional ethical committee approved the study.

### 2.3. Inclusion Criteria

1. Children in age group of below 12 years.
2. Peripheral smear or rapid malaria antigen test (RMAT) positive for* Plasmodium vivax* malaria.
3. Availability of a written informed consent.

### 2.4. Exclusion Criteria

1. Nonavailability of consent.
2. Peripheral smear positive for* P. falciparum* or positive for both* P. falciparum* and* P. vivax*.
3. Patient presenting with fever (malarial parasite negative on peripheral smear and/or RMAT negative) but treated empirically like malaria.

### 2.5. Diagnosis

The diagnosis and confirmation of species of* P. falciparum* and* P. vivax* malaria were established by thick and thin film of peripheral blood smear examination under oil immersion with Giemsa stain and RDT. The RDTs were based on detection of specific* Plasmodium* spp. lactate dehydrogenase and histidine-rich protein 2.

Categorization in severe and nonsevere malaria was done on the basis of WHO guidelines for severe malaria.

#### 2.5.1. Initial World Health Organization Criteria from 1990

- Cerebral malaria: unarousable coma nonattributable to any other cause, with a Glasgow Coma Scale score ≤9. Coma should persist for at least 30 min after generalized convulsions.
- Severe anemia: hematocrit <15% or hemoglobin <50 g/L in the presence of parasite count >10 000/*μ*L.
- Renal failure: urine output <12 mL/kg/24 hours in children and a serum creatinine >3.0 mg/dL despite adequate volume repletion.
- Pulmonary edema and acute respiratory distress syndrome.
- Hypoglycemia: whole blood glucose concentration <40 mg/dL.
- Circulatory collapse (algid malaria): systolic blood pressure <70 mmHg in patients >5 years of age (<50 mmHg in children aged 1.5 years), with cold clammy skin or a core-skin temperature difference >10°C.
- Abnormal bleeding and/or disseminated intravascular coagulation: spontaneous bleeding from gums, nose, and gastrointestinal tract, or laboratory evidence of disseminated intravascular coagulation.
- Repeated generalized convulsions: 3 convulsions observed within 24 hours.
- Acidemia/acidosis: arterial pH <7.25 or acidosis (plasma bicarbonate <15 mmol/L).
- Macroscopic hemoglobinuria: hemolysis not secondary to glucose-6-phosphate dehydrogenase deficiency.

#### 2.5.2. Added World Health Organization Criteria from 2000

- Impaired consciousness: arousable mental condition.
- Prostration or weakness.
- Hyperparasitemia: >5% parasitized erythrocytes or >250,000 parasites/*μ*L (in nonimmune individuals).
- Hyperpyrexia: core body temperature >40°C.
- Hyperbilirubinemia: total bilirubin >2.5 mg/dL.

### 2.6. Data Collection and Analysis

Data regarding patient age, sex, clinical presentation, investigations, and outcome were recorded. Patients were categorized in severe and nonsevere group based on WHO guidelines for classification of severe malaria. Chi square test was performed to test statistical significance of sex distribution in different age buckets. Prevalence of symptoms, signs, severity criteria, lab parameters, and their relation to mortality were studied.

### 2.7. Treatment

Patients were treated according to WHO guidelines for malaria treatment.

## 3. Results

### 3.1. Age Distribution of Cases

In this study most of cases were in the 6–12-year age group (see Table 1).

### 3.2. Sex Distribution of Cases

See Table 2.

### 3.3. Age and Sex Distribution

Overall male to female ratio was 1.94 : 1. Male to female ratio was 1 : 1 in <1-year age group; males outnumbered female cases in the age group from 6 to 12 years (male : female = 5 : 1) (see Table 3).

### 3.4. Clinical Features

See Table 4.

### 3.5. Distribution of Organomegaly

See Table 5.

### 3.6. Severe Disease

WHO has defined severity parameters for malaria, which were tested for our study in terms of their frequency and their association to the outcome in the form of mortality. 13 cases (26%) satisfied one or more criteria of WHO severity parameters and were classified as severe malaria. Clinical and laboratory profile of these cases are presented in Table 6.

### 3.7. Laboratory Parameters Not Included in WHO Criteria among Severe Malaria Cases and Outcome

See Table 7.

### 3.8. Outcome

Among the total of 50 cases studied 2 cases died with a case fatality rate of 4%.

Among the total of 50 cases studied 10 cases (20%) required ICU admission.

## 4. Discussion

Males were more affected than females, which is possibly due to increased outdoor activity and increased exposure to mosquitoes in males as compared to females. Kochar et al. found similar results in their study with 33.0% females affected among cases of* P. vivax* malaria. Age distribution among various age groups was 33.9% in 0–5 years, 30.1% in 5–10 years, and 30% in >10 years, which was almost similar in all age groups [6]. A study done in East Delhi studied population of 1 to 12 years which shows 59.7% males and 40.3% females having* P. vivax* malaria, as compared to our study which shows 69% males and 31% females having* P. vivax* malaria in similar age group of 1 to 12 years [7].

The most common presentation was fever, present in 48 out of total of 50 cases (96%).

High fever trends are evident in* P. vivax* disease even with lower parasitemia due to its recognized lower fever-threshold (around 100 infected RBCs/microliter) [8].

It was followed by pallor which was present in 31 cases (62%). Two common causes of anemia are increased hemolysis and decreased rate of erythrocyte production from bone marrow whereas the malnutrition and intestinal parasitic infections aggravate this problem in highly endemic areas. In a study about 50% of patients with* P. falciparum* and mixed infections were anemic while 29% of* P. vivax* infected cases had this abnormality [9].

Major confounding factors in the global analysis of anemia are the local contributors to this hematological complication such as iron-deficiency anemia [10].

None of the cases presented with renal impairment, hypoglycemia, jaundice, and hyperparasitemia.

The new WHO guidelines already point to hyperbilirubinemia (total bilirubin >3.0 mg/dL) as a weak marker of severity, unless it is followed by any other vital organ dysfunction [11].

Two cases died; both had pulmonary edema and bleeding and 1 case had impaired consciousness.

Major predictor of mortality among these predefined WHO severity criteria was pulmonary edema/ARDS with 100% mortality, whereas patients with severe anemia, circulatory collapse, and repeated generalized convulsions had 100% survival rate.

The statistical significance was not applicable as there were only 2 deaths among the study group.

Anstey et al. suggested that* P. vivax* patients are more likely to suffer from respiratory distress syndrome as they have more severe alveolar capillary dysfunction. Sequestration of* P. vivax* infected erythrocytes in the pulmonary microvasculature and greater inflammatory response to a given parasitic burden in* P. vivax* are probably responsible for this alveolar capillary dysfunction. Small airway obstruction, gas exchange alteration, increased phagocytic activity, and accumulation of pulmonary monocytes are the other suggested mechanisms for respiratory complications [12].

Case fatality rate in this study was 4%. Most of the earlier published literature consists of few death reports or small descriptive clinical series lacking denominators. Recent study from Papua, Indonesia, reported 1.6% and 2.2% case fatality rate caused by* P. vivax* and* P. falciparum*, respectively [6]. Case fatality rate in the studies above and this study thus stresses* P. vivax* infection to be almost equally serious in causing significant mortality in comparison to* P. falciparum*.

Among the laboratory parameters as mentioned in Table 7 major predictor of mortality emerged as leukopenia with 40% mortality followed by thrombocytopenia (platelet count <50,000/mm^3^).

Among the total of 50 cases studied 10 cases (20%) required ICU admission.

In a study done in Manaus a rough estimation of the risk of ICU admission per species showed similar relative risks for* P. vivax* (4.7/10,000 cases) and* P. falciparum* (5.5/10,000 cases). The study included all reported cases of malaria in children of 0–14 years of age in Manaus [13].

Our study population was not an ideal population representative and that may be the reason for high ICU admission rate in our study. Further study is needed to study the burden of* P. vivax* malaria on ICU facility in hospitalized patients so as to estimate the optimum requirement of facilities to maximize the patient care.

Limitations of our study were small sample size and existing confounding factors of different parameters, though the study shows the diverse clinical presentations of* P. vivax* malaria ranging from fever to cerebral malaria, ARDS, and pulmonary edema and also emphasizes the importance of severity of* P. vivax* malaria and there is a need for further studies to establish mortality and severity predictors specific to* P. vivax* malaria.

## 5. Conclusion

The present study highlights the epidemiology of* P. vivax* malaria in pediatric age group. Since* P. vivax* was considered a benign disease, there are scarce reports. The study stresses that* Plasmodium vivax* can result in severe disease and can no longer be considered a benign condition. The present study shows that some manifestations of WHO severity criteria were not seen in severe* P. vivax* malaria (renal impairment, hypoglycemia, jaundice, and hyperparasitemia), whereas leukopenia and thrombocytopenia which are not part of WHO severity criteria were frequently present and were associated with mortality. This implicates the need for separate severity parameters for* P. vivax* malaria. However, larger studies need to be undertaken to establish the specific severity parameters and poor prognostic indicators.

## Figures and Tables

**Table 1:** Age distribution of total cases (*N* = 50).

Age (years)	Number	Percentage
<1	8	16%
1 to 5	12	24%
6 to 12	30	60%

Total	50	100%

**Table 2:** Sex distribution of total cases (*N* = 50).

Sex	Number	Percentage
Female	17	34%
Male	33	66%

Total	50	100%

**Table 3:** Age and sex distribution of total cases (*N* = 50).

Age (years)		Sex		Total	*P* value (Chi square test)
		Female	Male		
<1^*^	Number	4	4	8	0.37
	%	50.0%	50.0%	100.0%	
1 to 5^*^	Number	8	4	12	
	%	66.7%	33.3%	100.0%	

6 to 12	Number	5	25	30	0.0002
	%	16.7%	83.3%	100.0%	

Total	Number	17	33	50	0.023
	%	34.0%	66.0%	100.0%	

^*^<1 year and 1 year to 5 years of age groups have been combined for Chi square test.

**Table 4:** Distribution of presentations in total cases (*N* = 50).

Features	Number of cases	Percentage
General		
Fever	48	96.0%
Pallor	31	62.0%
Edema	4	8.0%
Body ache	1	2.0%
Respiratory symptoms	18	36.0%
Cough	13	26.0%
Tachypnea	5	10.0%
Gastrointestinal	38	76.0%
Vomiting	24	48.0%
Abdominal pain	7	14.0%
Loose motions	4	8.0%
Abdominal distension	2	4.0%
Anorexia	1	2.0%
Jaundice	0	0.0%
Bleeding	5	10.0%
Bleeding (skin and mucocutaneous)	2	4.0%
Pulmonary bleeding with hematemesis	1	2.0%
Pulmonary hemorrhage	1	2.0%
Hematuria	1	2.0%
Central nervous system	18	36.0%
Headache	9	18.0%
Convulsions	4	8.0%
Altered sensorium	4	8.0%
Diplopia and dizziness	1	2.0%
Hypotonia	1	2.0%
Signs of raised intracranial tension	1	2.0%
Squint	1	2.0%
Imaging (chest X-ray, USG)		
Cardiomegaly	2	4.0%
Pleural effusion	2	4.0%
Pulmonary hemorrhage	1	2.0%
Pulmonary edema	1	2.0%
Ascites	1	2.0%

**Table 5:** Distribution of organomegaly among total cases (*N* = 50).

Organomegaly	Number	Percentage (%)
Hepatomegaly	5	10.0%
Splenomegaly	5	10.0%
Hepatosplenomegaly	25	50.0%
No hepatosplenomegaly	15	30.0%

Total	50	100.0%

**Table 6:** Distribution of WHO severity parameters and outcome.

WHO criteria for severe malaria	Number of patients with severe malaria	Percentage of severe malaria cases (%) (*N* = 13)	Number of cases that expired	Percentage of patients that died (%)
Severe anemia Hb < 5 mg/dL/PCV < 15%	3	23.0	0	0
Raised serum creatinine (>3.0 mg/dL)/ Urine output <12 mL/kg/24 hours	0	0	0	0
Pulmonary edema/ARDS	2	15.4	2	100.0
Abnormal bleeding	5	36.5	2	40.0
Hypoglycemia (<40 mg/dL)	0	0	0	0
Repeated generalized convulsions (≥3 in 24 hrs)	1	7.7	0	0
Circulatory collapse	2	15.4	0	0
Hyperparasitemia >5%	0	0	0	0
Serum bilirubin >2.5 mg/dL	0	0	0	0
Impaired consciousness/Glasgow Coma Scale <9	4	30.8	1	25.0

**Table 7:** Distribution of laboratory parameters not included in WHO criteria among severe malaria cases and outcome.

Sample number	Parameter	Range	Total number	Percentage of total cases	Died	
				(*N* = 50)	Number	% of cases
1	Total leucocyte count	Leukocytosis (>10000/mm^3^)	5	10.0%	0	—
		Leukopenia (<4000/mm^3^)	5	10.0%	2	40.0%
		Normal (4000–10000/mm^3^)	40	80.0%	0	—

2	Platelet counts (thousand/mm^3^)	>150	3	6.0%	0	—
		100 to 150	10	20.0%	0	—
		50 to 100	24	48.0%	0	—
		<50	13	26.0%	2	15.4%

3	Peripheral smear	Positive	49	98.0%	2	4.1%
		Negative	1	2.0%	0	—

4	Parasitic index	≤1%	45	90.0%	2	4.5%
		>1%	5	10.0%	0	—

5	RMAT	Positive	9	18.0%	0	—
		Negative	41	82.0%	2	4.9%

6	SGOT	Raised^#^	6	12.0%	0	—
		Normal	44	88.0%	2	4.6%

7	SGPT	Raised^#^	4	8.0%	0	—
		Normal	46	92.0%	2	4.4%

8	Serum bilirubin	Raised^#^	1	2.0%	0	—
		Normal	49	98.0%	2	4.1%

9	Serum creatinine	Raised (>1.0 mg/dL)	5	10.0%	1	20.0%
		<1.0 mg/dL	45	90.0%	1	2.2%

^#^Based on normal reference range for age and sex.
